# The natural history of *Calyptraeaaurita* (Reeve, 1859) from Southern Chile (Gastropoda, Calyptraeidae)

**DOI:** 10.3897/zookeys.798.25736

**Published:** 2018-11-21

**Authors:** Jorge Holtheuer, Cristian Aldea, Dirk Schories, Carlos S. Gallardo

**Affiliations:** 1 Instituto de Ciencias Marinas y Limnológicas, Universidad Austral de Chile, Casilla 567, Valdivia, Chile; 2 Asesorías Ambientales y Buceo Profesional, Punta Arenas, Chile; 3 Centro de Investigación GAIA Antártica y Departamento de Ciencias y Recursos Naturales, Universidad de Magallanes, Av. Bulnes 01855, Punta Arenas, Chile; 4 Forschungszentrum Jülich, Jülich, NRW, Germany

**Keywords:** Calyptraeid gastropod, external morphology, growth, radula, reproduction, shell, transplantation, Crecimiento, concha, Gasterópodos caliptreidos, morfología externa, rádula, reproducción, trasplante

## Abstract

Hard bottom communities of the Reloncaví Estuary and adjacent areas, Region de los Lagos, Chile (42°S), were studied between 2008 and 2011. All hard substrates between the lower intertidal and 25 m depth were dominated by the calyptraeid gastropods, *Crepipatelladilatata* and *C.fecunda*. Epibenthic coverage of the hard bottoms markedly decreased further down with the exception of vertical cliffs. In a depth range between 26 to 48 m repeatedly dense patches of another calyptraeid species, *Calyptraeaaurita* (Reeve, 1859), were observed. Densities reached up to 1475 individuals m^-2^ and covered up to 50 % of the rock surfaces. In shallower depths *C.aurita* was not present. However, despite its huge abundance, *C.aurita* has not been documented for more than 150 years in the southeastern Pacific, being described superficially by Reeve, through only shell characteristics. Here, we redescribe and compare it with other members of the family Calyptraeidae through characteristics of shell, radula, and soft parts, including also details of the egg mass and intracapsular development of their embryos. Males were mobile and females sessile. Shell size ranged from 6.6 to 12.4 mm for immature individuals, from 10.6 to 24.9 mm for males, 15.1 to 25.9 mm for intersex individuals, and from 21.0 to 39.6 mm for females. Up to three individuals stacked together were found, always presenting a female at the base with up to a maximum of two male individuals above. Laboratory studies demonstrated that *C.aurita* has an indirect larval development, liberating planktotrophic larvae with a bilobed ciliated velum into the water column. A transplantation experiment demonstrated that survival, growth, and reproduction of *C.aurita* is also possible in depths shallower than its normal distribution. The geographic distribution of *C.aurita*, was previously only known as being from Valparaíso (33°S) and is now extended down to the Reloncaví Sound (41°S).

## Abstract (es)

Comunidades de fondos duros del estuario de Reloncaví y áreas adyacentes, Región de los Lagos, Chile (42 ° S), fueron estudiados entre el 2008–2011. Todos los fondos duros entre la profundidad intermareal inferior y 25 m fueron dominados por los gasterópodos caliptreidos, *Crepipatelladilatata* y *C.fecunda*. La cobertura epibentónica de los fondos duros disminuyó notablemente a mayor profundidad, con la excepción de los acantilados verticales. En un rango de profundidades entre 26 y 48 m fueron observados parches repetidamente densos de una tercera especie de Calyptraeidae, *Calyptraeaaurita* (Reeve, 1859). La densidad alcanzó hasta 1475 ind. m^-2^ y una cobertura de hasta un 50 % de la superficie de las rocas. En profundidades menores *C.aurita* no estuvo presente. A pesar de su gran abundancia, *C.aurita* no se ha documentado desde hace más de 150 años en el Pacífico Suroriental, siendo descrita superficialmente por Reeve, solamente a través de las características de la concha. En este trabajo, nosotros redescribimos y comparamos esta especie con otros miembros de la familia Calyptraeidae a través de características de la concha, rádula y partes blandas, incluyendo algunos detalles de la masa de huevos y desarrollo intracapsular de sus embriones. Los machos fueron móviles y las hembras sésiles. El tamaño de la concha varía entre 6,6 y 12,4 mm para los individuos inmaduros, 10,6 a 24,9 mm para los machos, 15,1 a 25,9 mm para los individuos intersexo y desde 21,0 a 39,6 mm para las hembras. Fueron encontrados hasta tres individuos apilados juntos, presentando siempre una hembra en la base y hasta un máximo de dos individuos machos sobre ella. Los estudios de laboratorio demostraron que *C.aurita* tiene un desarrollo larval indirecto liberando una larva planctotrófica con un velo ciliado bilobulado en la columna de agua. Un experimento de trasplante demostró que la supervivencia, el crecimiento y la reproducción de *C.aurita* es posible en profundidades menores que su distribución normal. La distribución geográfica de *C.aurita*, anteriormente sólo se conocía desde Valparaíso (33°S) extendiéndose hasta el Seno de Reloncaví.

## Introduction

Gastropods with external shells belong to the most studied groups of all the marine fauna of Chile due to their economic importance (Valdovinos 1999, Castilla and Defeo 2001, Letelier et al. 2003, Aldea and Valdovinos 2005, Cárdenas et al. 2008). Nevertheless, the total number of species is still unknown, because many coastal regions are hard to access due to their remoteness or their extreme exposure to waves, which makes sampling difficult. Therefore new species records or redescriptions of species not found for decades are not surprising for the Chilean marine fauna (Sanamyan and Schories 2003, Lee et al. 2008, Sanamyan et al. 2010). Less attention has been paid to those species that are not used for human consumption. In this sense, only three calyptraeid gastropods have been studied intensively during the last decade (Brown and Olivares 1996, Chaparro et al. 1999, Chaparro et al. 2001a, Chaparro et al. 2001b, Chaparro and Flores 2002, Navarro and Chaparro 2002, Chaparro et al. 2005, Daguin et al. 2007, Chaparro et al. 2008, Brante et al, 2011). Two of these calyptraeid species, *Crepipatelladilatata* (Lamarck, 1822) and *Crepipatellaperuviana* (Lamarck, 1822) [named as *C.fecunda* (Gallardo 1979b) before Véliz et al. (2012)], are among the most common species in the Northern Chilean fjord and channels ecoregion of southern Chile and adjacent estuaries, often covering rocks, boulders and mussels up to a depth of 25 m. Despite their abundance in the subtidal zone of the northern fjords of southern Chile, the presence of a third species (*Calyptraeaaurita*) with a vertical distribution below the other ones was unknown up until now and is really surprising. (1) The Reloncaví Sound has been studied intensively on several previous expeditions, including the Swedish Lund expedition (1948–1949) which produced several monographs of different taxa (Brattström and Dahl 1951, Brattström and Johannsen 1983) and (2) the species was found in high abundance in an area easy to access by SCUBA divers.

Among the 18 known species of Calyptraeidae of the continental coast of Chile (Valdovinos 1999), the case of the species *C.aurita* is unique and no documented in that report. The species was only described by shell morphology of individuals dredged in the vicinity of Valparaíso. Besides the short species description provided by Reeve (1859) only two small illustrations showing both sides of a shell were available. Various authors made an inventory of the Chilean malacofauna, however no one mentioned or found this species again along the Chilean coast or included it in databases or check–lists (Guzmán et al. 1998, Gutt et al. 2003, Aldea and Valdovinos 2005, Osorio et al. 2006, Ramajo and Osorio 2010). Due to its stable population presence during 2008–2012 we were able to add new biological and ecological data to the species.

A major part of taxonomic studies of mollusks is still based on shell characteristics of type specimens (Guzmán et al. 1998). Often no associated additional biological data or information about their geographical distribution exists. Consequently, taxonomic classification underlies continuous modifications due to the apparition of new characteristics. This, at the same time, produces continuous modifications in the taxonomy of many taxa due to the apparition of new characteristics and the evaluation of the plasticity of different nominal species within a specific group (Ponder and Lindberg 1997, Letelier et al. 2003). A good example is sibling species, which may be indistinguishable by appearance, but nevertheless are reproductively isolated from one another (Gallardo 1979b).

The family Calyptraeidae counts approximately 139 living species (123 valid) in total (MolluscaBase 2018), of which nearly 40 have been reported from the Eastern Pacific (Paredes and Cardoso 2007). All species of the family are protandrous hermaphrodites (Brown 1989) and sedentary filter–feeders (Collin 2003a, Collin et al. 2007). All species of the genus *Calyptraea* have a patelliform shell that retains some remains of spiral coiling (Simone 2002). The septum is modified into a curved plate. The presence of the genus *Calyptraea* has been reported since the Paleocene and Oligocene for the Pacific Ocean and since the Eocene for the Northern Atlantic (Saul and Squires 2008). Morphological plasticity, in response to environmental factors and a relatively simple morphology within the Calyptraeidae, resulted in a huge variation of the shell form. Therefore the relatively high number of synonyms for each species is not surprising. Today, additional characteristics like internal anatomy and DNA sequencing (Simone 2002, Daguin et al. 2007) are often used, where shell characteristics and larval development are not sufficient. In calyptraeid gastropods embryonic and larval development present an additional tool to distinguish morphologically very similar species like *Crepipatelladilatata* and *C.peruviana* (Coe 1938, Coe 1949, Gallardo 1977, Gallardo 1979b). Our study provides information on the encapsulated development of the embryos of *C.aurita* as well as information on its subtidal distribution.

## Materials and methods

### The study area

**Figure 1.**
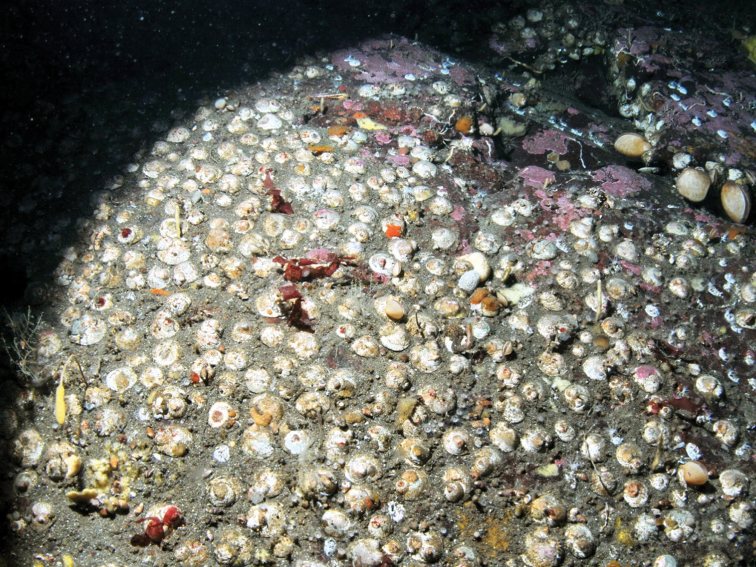
High densities of *Calyptraeaaurita* in a depth of 34 m mean tide level at Caleta la Arena (Reloncaví Sound).

**Figure 2.**
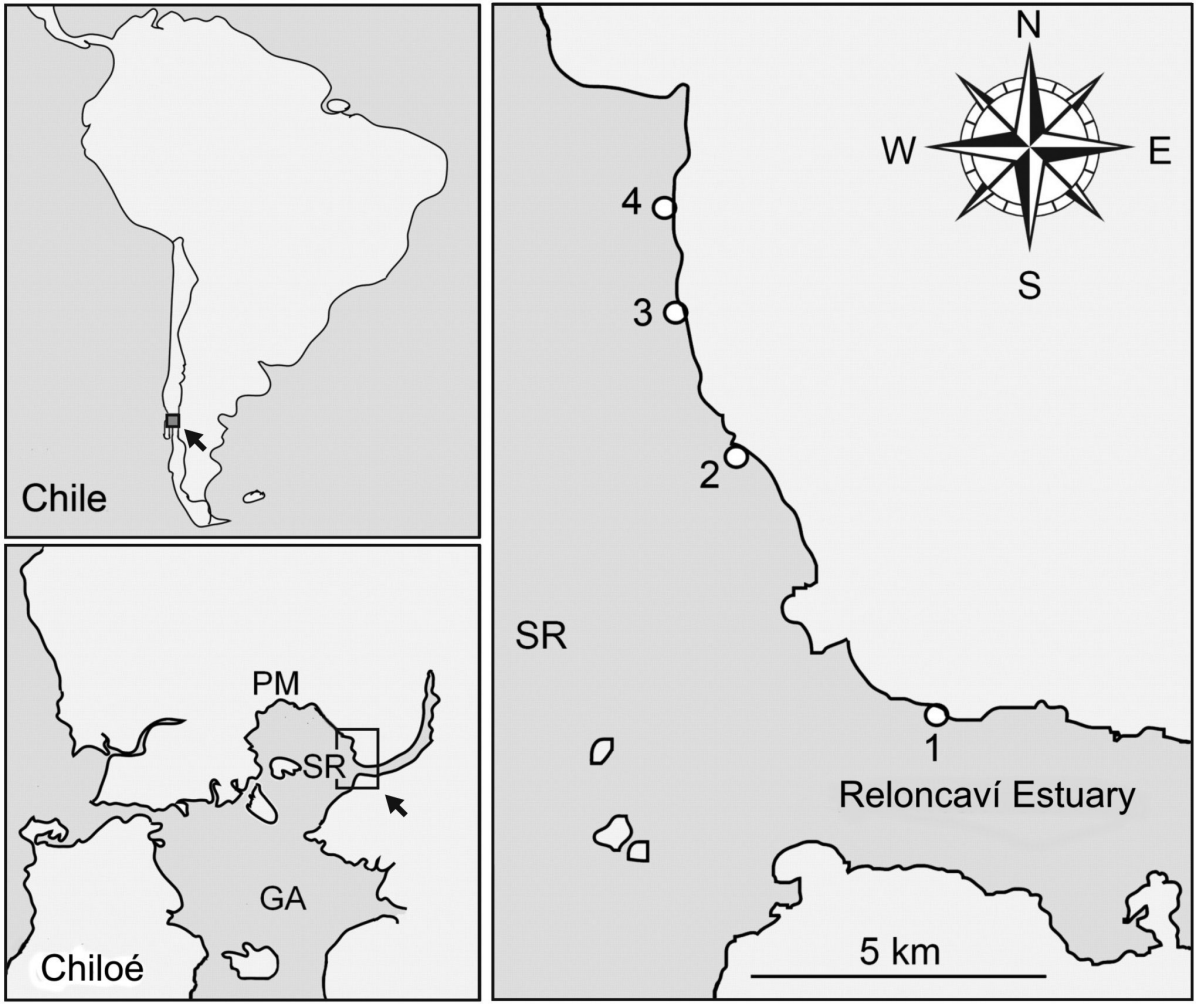
The study was carried out in Southern Chile (upper left) in the Reloncaví Sound (lower left) in its eastern part (right). **1** Reloncaví Estuary **2** Caleta Yerbas Buenas **3** Caleta Gutiérrez **4** Caleta Chaicas; PM = Puerto Montt; GA = Golfo de Ancud; SR = Reloncaví Sound.

In May 2008 we noticed for the first time the presence of *Calyptraeaaurita* in depths below 30 m near Caleta Yerbas Buenas (41°40'20"S, 72°39'24"W), Reloncaví Sound, Chile (Figure 1). Yerbas Buenas is situated 32 km southeast of the city of Puerto Montt (Region de los Lagos) (Figure 2). Large diurnal temperature fluctuations of the upper water column, measured during summer time, are not detectable below 20 m depth, where water temperature varies only between 10 °C and 13 °C, salinity is constant at approx. 35 PSU. Mean tidal range is approximately 4 m with 2 m at neap and more than 6 m at spring tides. All animals collected for laboratory studies were taken from Caleta Yerbas Buenas.

Three additional sites were chosen to study the possible presence of *C.aurita* in the vicinity of the first place: (1) Reloncaví Estuary: 41°42'33.13"S, 72°37'30.95"W, (2) Caleta Gutiérrez, 41°39'15.48"S, 72°40'1.20"W, and (3) Caleta Chaicas: 41°38'17.78"S, 72°40'10.94"W (Figure 2).

### Coverage, depth distribution, size, and abundance

The distribution and coverage of *C.aurita* were studied using transect photographs along a depth gradient and analyzed with the Windows–based software CPCe 3.6 (Kohler and Gill 2006). Five images, taken with a Nikon D70s, were taken and analyzed for each depth of 5 m, 10 m, 15 m, 20 m, 25 m and 30 m in a total horizontal distance of 15 m. A total of 100 specified numbers of points were distributed uniformly on each transect image and the individuals underlying the points were counted. Each photo frame had a size of 26 × 30 cm. The camera was fixed on a rack to guarantee that all images were taken from the same distance to the rock and to avoid inclination of the camera in relation to the rock. The abundance of the *C.aurita* within each frame was counted with the software ImageJ 1.41. Additional images were taken from patches of *C.aurita* in depths between 25 m to 48 m to estimate its maximum abundance and density.

In October 2009 a total of 190 individuals were sampled from four quadrants in order to estimate shell length distribution, sex ratio and the relationship between dry tissue biomass and shell length. The samples were fixed in 4% formalin–seawater after sampling. Shell sizes were measured with a digital caliper and sexual maturity status was registered for each individual. Afterwards, tissue biomass was separated from the shell, rinsed with freshwater and dried for 48 h at 60 °C.

### Taxonomy

Ten specimens were used for the species description. These specimens are deposited in the collections of mollusks, Laboratory of Malacology, Zoology of Invertebrates of the National Museum of Natural History (**MNHNCL**), Santiago de Chile, and the Instituto de Zoología, Universidad Austral de Chile (**IZUA-UACH**), Valdivia, Chile (Table 1). Additionally, images of four syntypes have been examined from the collection of the Natural History Museum of London (**NHMUK**). Radulae from three individuals (approx. 20 mm) were obtained by dissection under stereomicroscope and washed in sodium hypochlorite (5 %) in distilled water for five min to remove extraneous tissue.

**Table 1.** Shell morphometrics of *Calyptraeaaurita*. All measurements in mm.

Specimen	Total length	Width	Height	Sex
MNHNCL 7570	32.92	31.89	11.64	female
MNHNCL 7571	28.5	28.05	10.41	female
MNHNCL 7572	16.2	17.04	6.64	male
MNHNCL 7573	17.14	16.44	7.66	male
MNHNCL 7574	37.29	34.66	13.15	female
IZUA-UACH Mg 501	21.51	21.48	9.25	male
IZUA-UACH Mg 502	20.88	19.69	7.83	male
IZUA-UACH Mg 503	29.59	26.36	12.65	female
IZUA-UACH Mg 504	25.78	25.41	11.45	female
IZUA-UACH Mg 505	33.84	33.65	12.27	female

### Reproduction

Scuba divers in the field collected a total of 38 females with egg capsules in their mantle cavity, removing them carefully from the rock. Females without egg capsules were reattached by the divers to the rock. Back at the beach, the collected individuals reattached quickly to acrylic plates stored in coolers with 80 L saltwater and were transferred to the laboratory. Up to ten individuals were allowed to fix on a single acrylic plate, 40 × 45 cm in size. In the laboratory the plates with the attached individuals were transferred to 250 L tanks with permanent water flow and exchange. We used unfiltered seawater for the experiment and did not supply additional food. Intracapsular development was observed every three days with inverted light microscopy.

The different embryonic stages up to the liberation of the larvae are described using the criteria of Wyatt (1960), Zaixso (1973), Gallardo (1977), Penchaszadeh (1976), and Véliz et al. (2003).

### Transplantation experiment

An experiment was conducted to determine whether growth and reproduction is possible in shallower depths than those found in the field. Specimens collected at 30 m depth at Yerbas Buenas were marked with numbers on the shore and allowed to reattach onto transparent acrylic plates (25 × 40 cm, 20 individuals per plate). Each animal was measured and the plates with the animals on them were placed vertically at 10 and 20 m depth (four plates in each depth). After 165 and 326 days each animal’s length was measured to monitor its growth. Growth percentage was estimated based on the initial size compared with the final size at the end of the experiment. A t–test was used to compare growth after 165 days in 10 m and 20 m depth. The heavy loss of individuals during the course of time did not allow for the data measured at the end of the experiment to be included in the statistics.

## Systematics

### Phylum Mollusca Linnaeus, 1758

#### Class Gastropoda Cuvier, 1795

##### Order Littorinimorpha Golikov & Starobogatov, 1975

###### Family Calyptraeidae Lamarck, 1809

####### Genus *Calyptraea* Lamarck, 1799

######## Calyptraea aurita

Taxon classificationAnimaliaLittorinimorphaCalyptraeidae

(Reeve, 1859)

Calyptraea striata (non Say, 1826): Broderip 1834: 38; Broderip 1835: 202, pl. 28, fig. 6.
Crucibulum auritum Reeve, 1859: sp. 17, pl. 6, fig. 17a, b; Tryon 1886: 118 (in part), pl. 32, figs 32, 33.

######### Type material.

[*Crucibulumauritum*] is housed at NHMUK 197798.

######### Material examined.

MNHNCL 7570 (female), MNHNCL 7571–7574 and MZUA–UACH 501–505, all specimens from Caleta Yerbas Buenas, 41°40'20"S, 72°39'24"W. All coll. Jorge Holtheuer and Dirk Schories.

######### Description.

**Figure 3.**
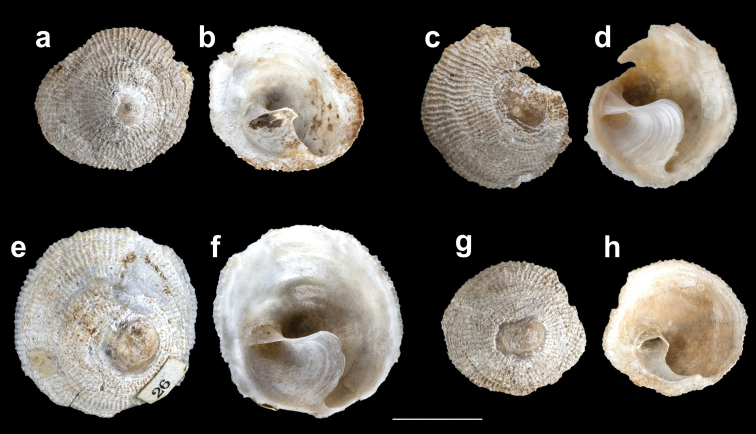
*Calyptraeaaurita*. NHMUK 197798, syntypes of *Crucibulumauritum* Reeve, 1859. **a, c, e, g** dorsal view **b, d, f, h** ventral view. Scale bar: 1 cm.

**Figure 4.**
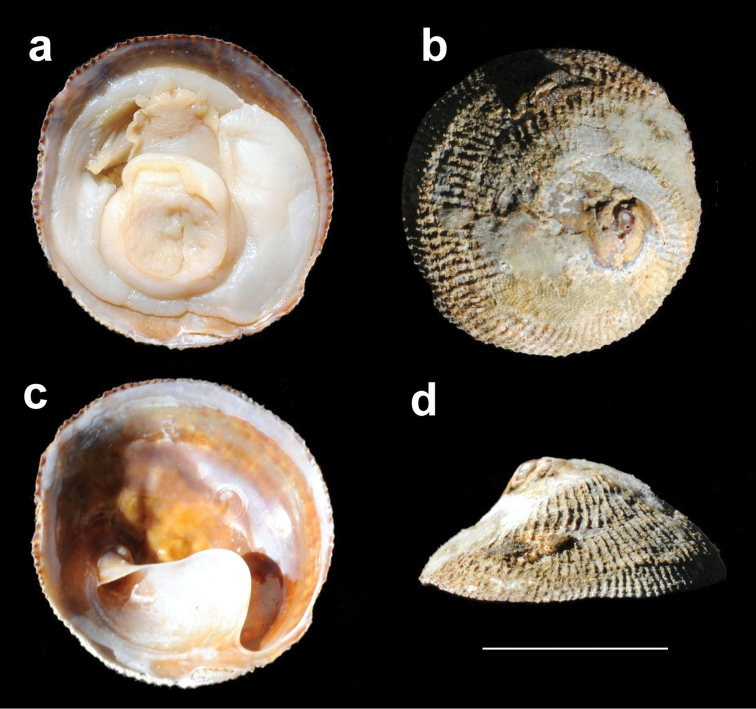
*Calyptraeaaurita*. MNHNCL 7570, female specimen. **a** ventral view of shell and head–foot **b** dorsal view **c** ventral view **d** lateral view. Scale bar: 2 cm.

**Figure 5.**
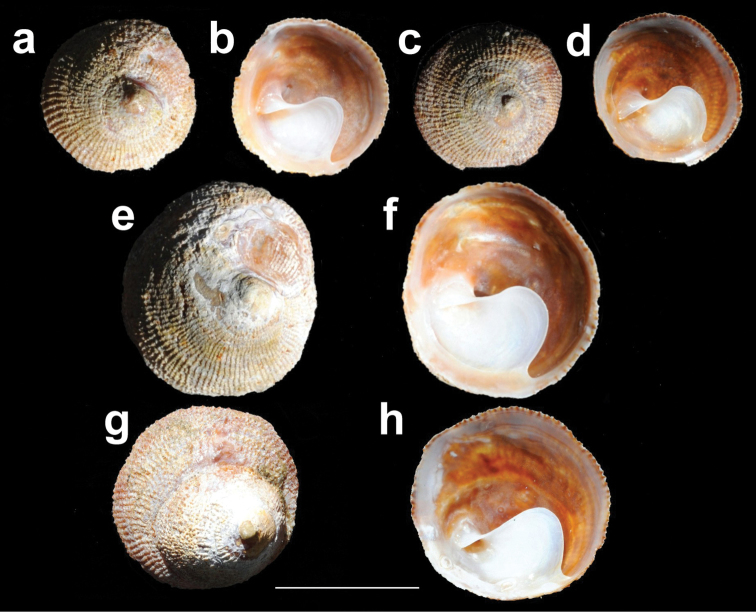
Shells of *Calyptraeaaurita*. **a**IZUA-UACH Mg 501 dorsal view **b**, ventral view **c**IZUA-UACH Mg 505 dorsal view **d** ventral view **e**IZUA-UACH Mg 502 dorsal view **f** ventral view **g**IZUA-UACH Mg 504 dorsal view **h** ventral view. Scale bar: 2 cm.

**Figure 6.**
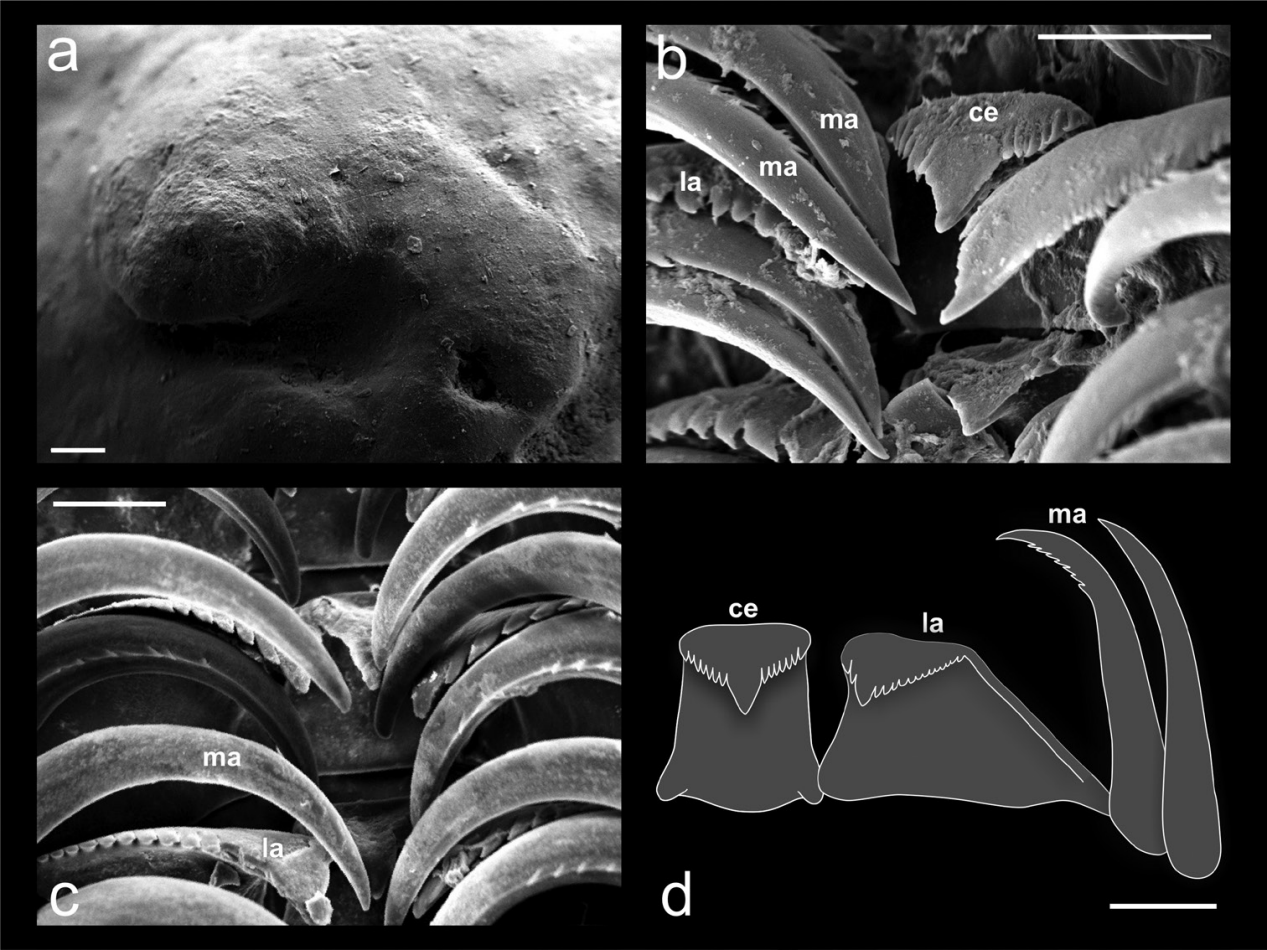
Protoconch and radula of *Calyptraeaaurita*. **a** protoconch in apical view **b** SEM showing teeth in folded position **c** SEM showing teeth in folded position **d** radular structure. Abbreviations: ce: central tooth; la: lateral tooth; ma: marginal tooth. Scale bar: 100 µm (**a**), 50 µm (**b, c, d**).

*Shell* (Figures 3a–h, 4b–d, 5a–h): Limpet–like, circular, conic, with spiral septum in center and right of ventral surface. The shell externally is usually opaque white and internally dark brown porcelaneous. The apex is small, sub–central; protoconch apparently smooth, with a total diameter difficult to measure because the protoconch–teleoconch boundary is not evident, but may have ~500 μm (Figure 6a). Sculpture has ~ 70 to 80 fine radial ribs, of uniform size, aligned concentrically. Inner surface without visible muscle scars. Septum incompletely conical (opened anteriorly), situated obliquely, from shell apex to posterior. Shell size ranged according to the sexual phases, being from 6.6 to 12.4 mm for immature individuals, from 10.6 to 24.9 mm for males, 15.1 to 25.9 mm for intersex individuals and from 21.0 to 39.6 mm for females (Table 2). Septum (Figures 3d, f, 4c, 5b, d, f, h) white brilliant color, beginning in a spiral conic curve and ending in a wide platform curved to the left side of the specimen (seen from below). Fine growth lines are visible on the septum.

**Table 2.** Shell length in mm of *Calyptreaaurita* sampled randomly at Yerbas Buenas. N = number of individuals sampled; SD = Standard Deviation.

	N	Min	Max	mean	SD
immature	9	6.6	12.5	9.1	1.7
Male	82	10.6	24.9	17.6	3.7
intersex	6	15.1	25.9	19.2	4.2
Female	83	21.0	39.6	31.7	3.5

*Radula* (Figure 6b, c, d): Radula with ca. 30 rows. Rachidian tooth broad, approx. 15 cusps, central cusp more elongated than secondary cusps (Figure 6b, d); lateral teeth curved inwards, with approx. 16 sharp cusps, two cusp on inside and ca. 13 gradually decreasing towards lateral on outside of main cusp (Figure 6c, d); marginal teeth long, tall, slender, with approx. seven sharp, sub–terminal cusps in inner edge (Figure 6b, d); outer marginal weakly narrower than inner marginal teeth.

**Figure 7.**
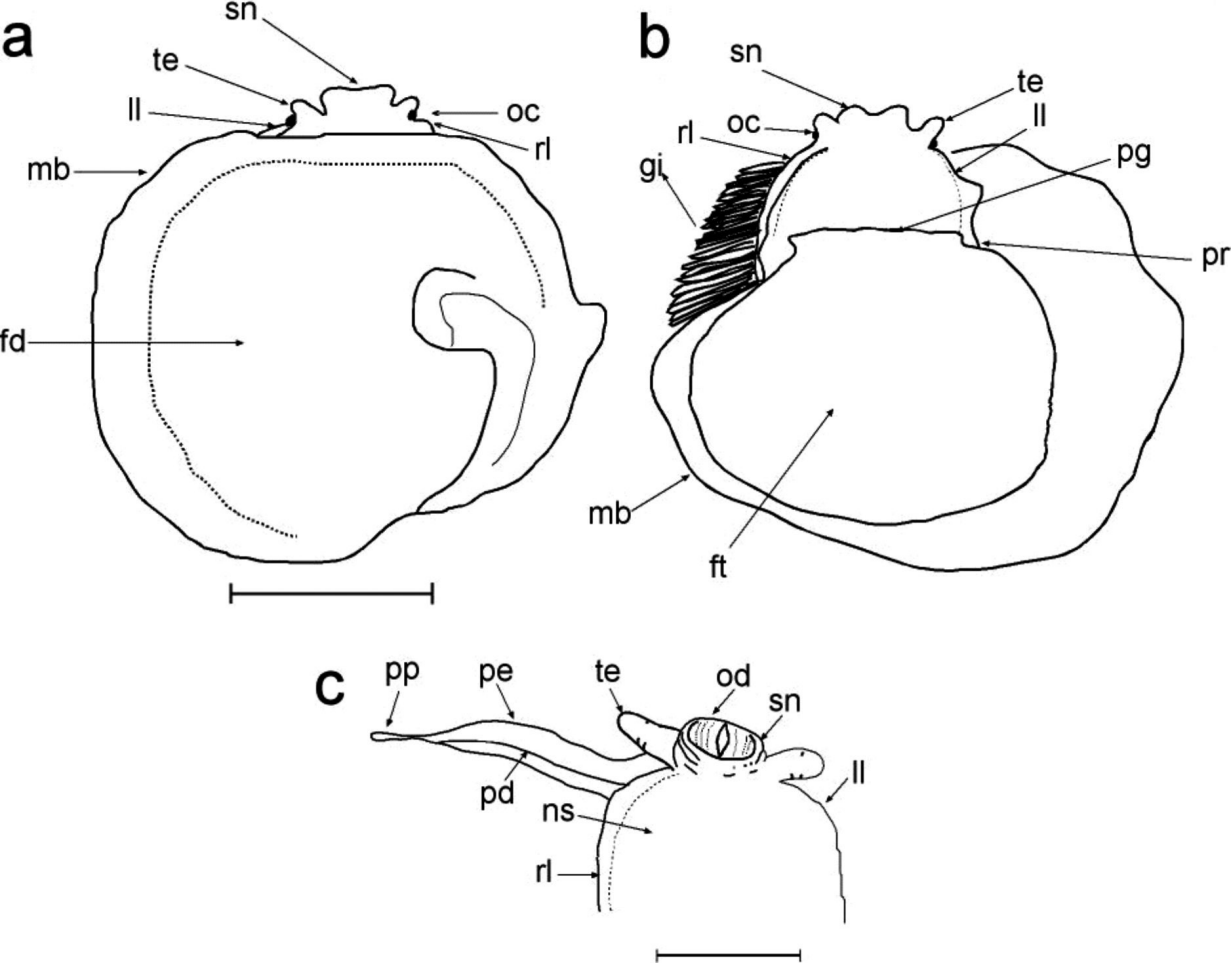
External morphology of *Calyptraeaaurita*. **a** female, without shell and whole, dorsal view **b** same animal, whole, ventral view **c** male, foot removed, ventral view. Abbreviations: fd: dorsal surface of foot; ft: foot; gi: gill; ll: left lateral expansion (flan); mb: mantle border; ns: neck “sole”; pd: penis sperm groove; pe: penis; pg: pedal gland anterior furrow; pp: penis papilla; pr: propodium; rl: right lateral expansion (flan); sn: snout–proboscis; te: cephalic tentacle. Scale bar: 10 mm (**a, b**), 5 mm (**c**).

*Head–foot* (Figures 4a, 7a, b): Head and neck regions somewhat similar to the other *Calyptraea* species, including neck ventral surface and flaps, penis present in all specimens behind right tentacle, but it is reduced or missing in females. Snout–proboscis very well developed, cephalic tentacles simple, eyes dark and small, located on the tentacle basis at lateral outward position. Foot similar to that of other *Calyptraea* species, with planar, dorso–ventrally flattened sole compressed by shell septum. Mantle, as in *Calyptraea*, attached to dorsal surface of foot sole and extending beyond its posterior and lateral borders.

*Mantle* (Figures 4a, 7a, b): Mantle border very broad, including region surrounding foot, occupying 90% of pallial cavity. Pallial cavity conical and curved, begins just inside shell septum. Pallial aperture proportionally small, if animal compared with a clock, this aperture begins at 10 and finishes at 6 o’clock.

*Gill*: typical to those of *Calyptraea*, occupying most of inner pallial space, inserts all along left and anterior pallial margins. Gill filaments also similar to those of *Calyptraea*, with very long (Figure 7b), rigid rod, mainly of the apical region. Gill posterior end just in posterior end of cavity; gill anterior end in central region of pallial aperture.

*Male* (Figure 7c): Only small specimens (up to 10.58 mm) are functional males, all mobile. Penis is very long (approx. three times head length), originating dorsally and extending to right tentacle. Papilla on penis tip, very long, approx. 1/3 of penis length. The penis sperm groove runs along middle region of the ventral surface of penis. The male of *Calyptreaaurita* is always attached onto a female, and is never found directly attached on primary substrate.

**Figure 8.**
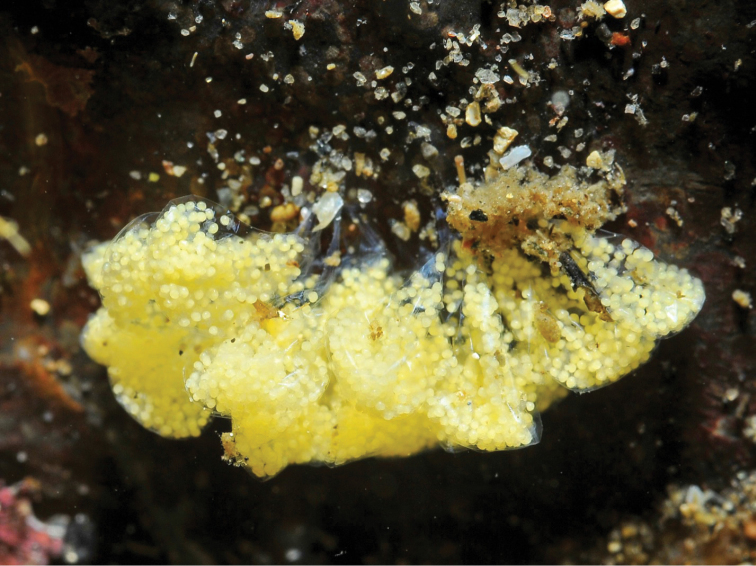
Egg capsules of *Calyptraeaaurita*. Female was removed by the diver, 31 January 2009.

*Female* (Figures 4a–d, 7a–c): Very similar to other *Calyptraea* species, the female is sessile. Only specimens larger than 20.98 mm were present in our material. During field work the presence of egg capsules in the pallial cavity of the females could be seen. The females always settle on the rock surface where they attach and protect their egg capsules up to larvae release (Figure 8).

######### Reproduction and development.

*Calyptraeaaurita* (Reeve, 1859) is a protandric hermaphrodite producing a maximum of 16 egg capsules per female which contain an average of 119 eggs each (Figure 8). Up to three individuals were observed stacked together, always with a female at the base and up to a maximum of two male individuals on her shell. Brooding was observed during the months March (38 of 100 females studied below water), April (38 of 100), August (44 of 55), September (150 of 199), October (36 of 50) and December (10 of 19). In May 2009 none of 100 observed females were brooding. Weather conditions did not allow for the verification of brooding during the other months.

The females of *Calyptraeaaurita* deposit their eggs in thin–walled brooded capsules directly attached to hard substrates. These capsules have a triangular, flattened morphology and are fixed with a fine stem to the substrate. The eggs are concentrated at the distal end of the sac embedded in an uncoloured liquid. All eggs are able to develop into planktonic veliger stages which are liberated into the water column. The veliger has a bilobed ciliated and pigmented velum and two small black–coloured eyes between the velar lobes, a circular mouth, and a transparent protoconch. The mean initial egg size is ca. 150 μm and the size of the veliger, when liberated into the water column is ca. 300 μm. The intracapsular development up to the larval release took ca. 42 days in the laboratory.

**Figure 9.**
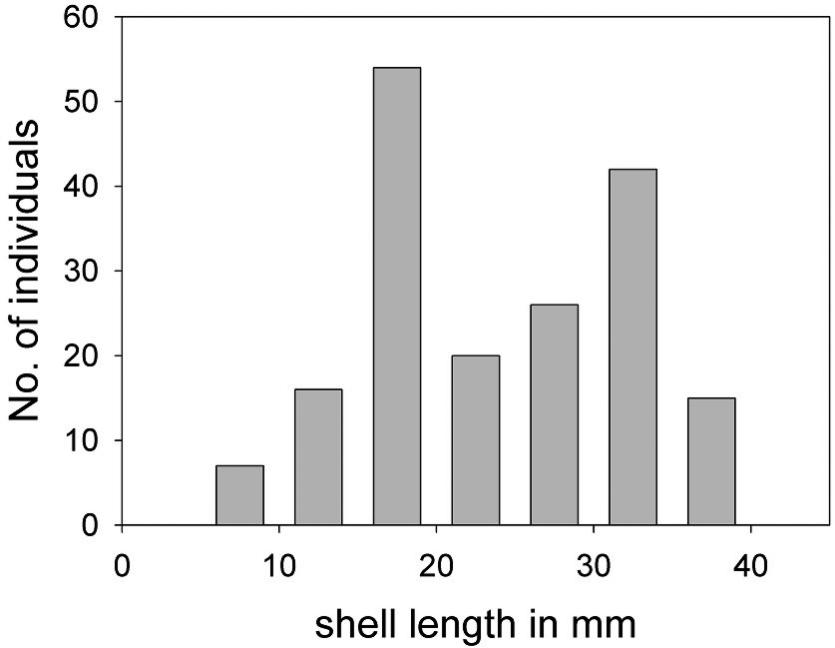
Size distribution of *Calyptraeaaurita* (n = 180).

*Size*: A total of 180 individuals were collected in October 2010 in 30 m depth. Shell length, height, and width were measured. Shell length distribution was two peaked, the first peak corresponded to males and intersex individuals and the second peak to females (Figure 9). Mean shell length was 23.9 ± 8.3 mm, mean width 23.6 ± 8.5 mm and mean height 9.1 ± 2.7 mm. Shell width to length relation was close to 1 (0.99 ± 0.10) and shell height to length relation was 0.39 ± 0.05.

######### Symbionts.

Four samples with 38 to 77 females were taken in October 2010 and were checked underwater for the presence of the pinnotherid decapod *Calyptraeotherespolitus* (Smith, 1870). A total of 4.5 ± 1.3 % of females were infested by *C.politus*. None of the infested females deposited eggs.

######### Distribution and habitat.

*Calyptraeaaurita* occurs at Valparaíso at depth of 82–110 m (Broderip 1834). In this study it was found exclusively on hard substrates in the Reloncaví Sound between 26 to 48 m depth showing a patchy distribution. The species was present in three of four study sites using vertical transects down to 30 m depth. 37.6 ± 14.4 % of the rocks were covered by *C.aurita* at the location Caleta Yerbas Buenas at 30 m depth. This represented a density of 743 ± 307 ind. m^-2^. The highest observed density was 1475 ind m^-2^ covering 50 % of the primary substrate. Coverage at the locations Caleta Gutiérrez and the Reloncaví Estuary were low with 1.8 ± 2.7 and 0.8 ± 1.8 % at the same depth. In none of the locations *C.aurita* was present along transects in shallower depths (5 to 25 m).

######### Transplantation experiment.

**Figure 10.**
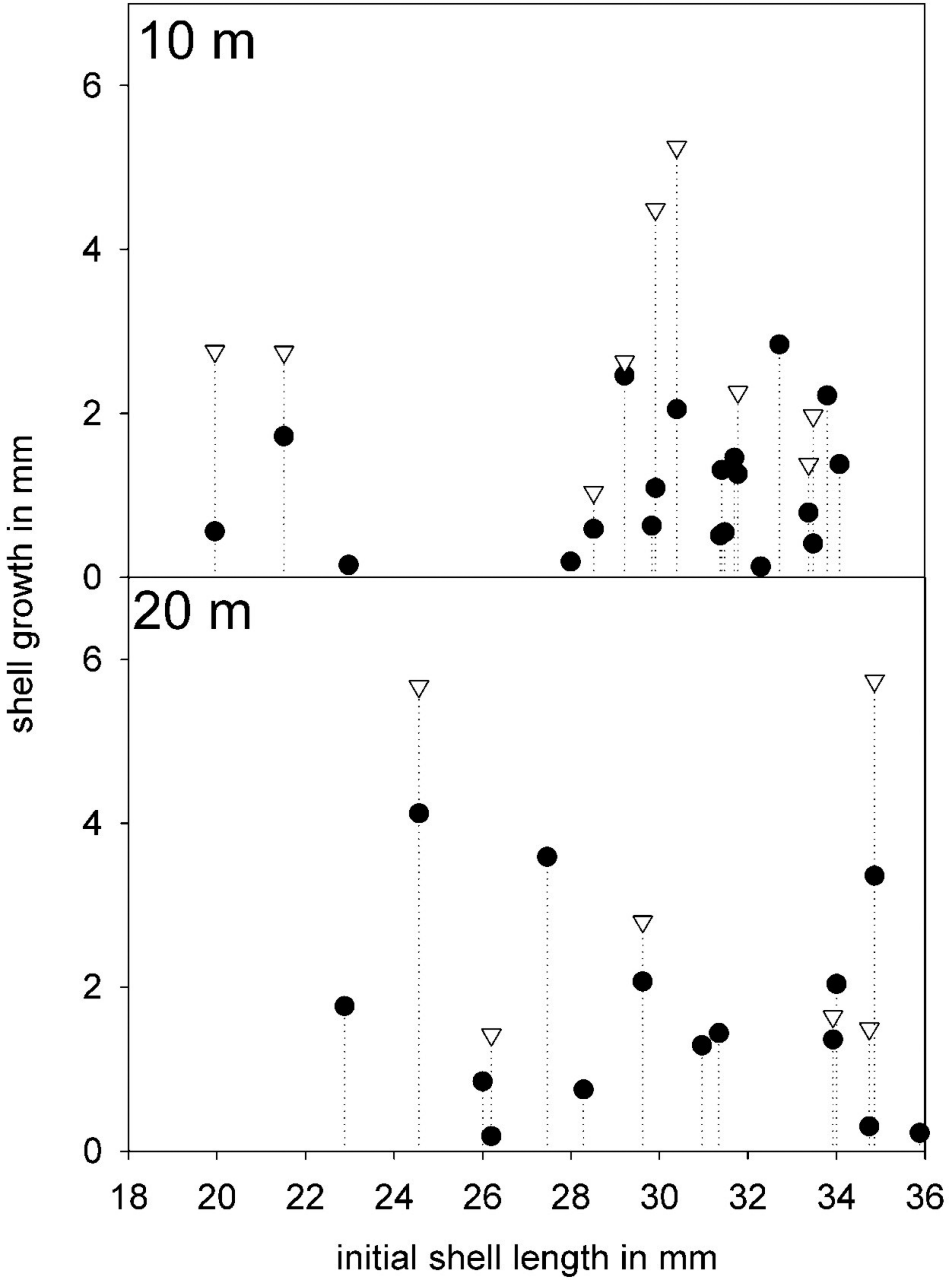
Individual growth rates of *Calyptraeaaurita* after 165 (black circles) and 326 days (triangles) in 10 m and 20 m depth.

The experiment was realized for a total time span of 326 days. Several individuals got lost during transport from the experimental depth to the shore, died during course of time, or did not reattach once unintentionally detached from the acrylic plate. Nevertheless all remaining individuals grew several mm in both depths (Figure 10). Additionally some individuals deposited egg capsules, which were visible from the reverse of the acrylic plate. A t–test did not show differences between the growth rates in 10 and 20 m depth after 165 days, t(32) = t–1.555, p = 0.13. Mean growth rate in 10 m depth was 1.11 mm (n = 20) and 1.68 mm (n = 14) in 20 m depth, respectively.

## Discussion

### Taxonomic remarks

Broderip (1834, p. 38) described *Calyptraeastriata*, from Valparaíso, briefly indicating the same characteristics as the currently valid species *C.aurita*. However, *C.striata* Say, 1826 was previously described for the northwestern Atlantic. This last species is currently valid as *Crucibulumstriatum* (Say, 1826). Then the same author again describes and figures the species (Broderip 1835, p. 202, pl. 28, fig. 6). Later Reeve (1859) described *Calyptraeaaurita*, to report *Calyptraeastriata* Broderip (not of Say) as a junior synonym, noting that this species is less conoid than the valid *Crucibulumstriatum* (Say, 1826) and differently striated; the internal septum reaches nearly to the margin. The original description and the illustrations made by *Calyptraeaaurita* fit best with the material collected in the Reloncaví Sound: Shell dirty white, approximately circular, subconic, subturbinate, numerous corrugated longitudinal stripes, brownish–yellow color inside. Diameter 2.12 cm, height 0.76 cm.

Only two valid species of *Calyptraea* (s. s.) are considered along the coasts of the southeastern Pacific: *C.aurita* (Reeve, 1859), distributed in central and south Chile, and *C.mamillaris* Broderip, 1834, from Baja California to Peru. *Calyptraeamamillaris* differs externally from *C.aurita* in having only growth striations and external and internal white coloration and the apex located in the central part of the shell (see Collin 2003a, Paredes and Cardoso 2007).

*Calyptraeaaurita* was reported by Barnard (1963) for the South African coasts, providing the description of the shell and radula of the species. Nevertheless, Kilburn (1980) re–established Barnard’s (1963) record as a new species, *C.barnardi* Kilburn, 1980, arguing that having examined four syntypes of *C.aurita* (British Museum Natural History, No. 197798), the apex is markedly more eccentric and the sculpture consists of wavy, granular radial ribs, instead of concentric rings of fine scales as in the new South African taxon. However, Rolan (2005) in his discussion of another South African species, kept *C.aurita* valid for South Africa, without mentioning *C.barnardi*. Considering the abovementioned, there are obvious differences between *C.barnardi* and *C.aurita*. Apart from the characteristics of the shell mentioned by Kilburn (1980), the radula of *C.barnardi* drawn by Barnard (1963) has a rachidian tooth with five cusps and laterals teeth with ca. 13 cusps, instead of the rachidian with 15 cusps and laterals with 16 cusps of *C.aurita*.

According MolluscaBase (2018), other species of *Calyptraea* described from the Southeastern Pacific and Southwestern Atlantic are:

- Calyptraeaaraucana Lesson, 1830 (taxon inquirendum), from Concepción Bay, a species with marked radial ribs, that was established as junior synonym of Trochitatrochiformis (Born, 1778) by Tryon (1886) and Rehder (1943).
- Calyptraeapallida Broderip, 1834 (taxon inquirendum), from Falkland Islands, is a junior synonym of Crepipatelladilatata (Lamarck, 1822) according Tryon (1886) and Hoagland (1977), although Veliz et al. (2012) cited it as species with “status unclear”. However, according description and figures provided by Broderip (1835: 204, pl. 29, fig. 3), the generic position of this species certainly should be in Crepidula or Crepipatella.
- Calyptraeastrigata Broderip, 1834 (taxon inquirendum), from Valparaíso, was cited first as a junior synonym of Crepipatelladorsata (Broderip, 1834) by Tryon (1886) and was cited later as a junior synonym of Crepipatelladilatata (Lamarck, 1822) by Hoagland (1977), but Veliz et al. (2012) cited it as species with “status unclear, possibly a valid species”. In the same way that C.pallida, this species should belong to the genus Crepidula or Crepipatella (see Broderip 1835: 203, pl. 28, fig. 12, Veliz et al. 2012: fig. 6A).
- Calyptraeachiliensis Lesson, 1830 (nomen dubium), from Concepción, is an unfigured and undetermined species with types probably lost (see Tryon 1886, Hoagland 1977, Veliz et al. 2012).
- Calyptraeadepressa Lesson, 1830 (nomen dubium, invalid), from Concepción Bay, is a junior synonym of Crepipatelladilatata (Lamarck, 1822), but some specimens of the lot may be belong to Crepipatellaperuviana (Lamarck, 1822) (see Veliz et al. 2012).

On the other hand, the species *Calyptraeaintermedia* d’Orbigny, 1839, was described from the coasts of Peru, and still awaits confirmation of validation. Until today it has not been re-recorded, except for the reports of Tryon (1886) and Keen (1966). According to the description by its author, this species is white, conical, thin, diaphanous, and very depressed, with marked radial coasts very distant and not very prominent. According to Tryon (1886) it could be an aberrant juvenile form of *Trochitatrochiformis* (Born, 1778).

The geographical distribution of other calyptraeid species, *Trochitapileus* (Lamarck, 1822) and *T.pileolus* (d’Orbigny, 1841), overlaps with *C.aurita* (Forcelli 2000, Collin 2003a), but shell characteristics are distinct (see Pastorino and Urteaga 2012).

### Biological and ecological aspects

Reproduction of *Calyptraeaaurita* occurs during most of the year with the exception of the winter months. In June (austral winter) no brooding was observed, but data for July and August are lacking. In general, calyptraeid species are known to vary in brooding season, some species like *Crepidulaadunca* brood throughout the year whereas others like *C.lingulata* brood only throughout the summer months (Collin 2000, Henry et al. 2010). *C.aurita* liberates approx. 2000 veliger larvae, because virtually all embryos hatch. This number is low compared to *Crepidulafornicata* but comparable to *C.lingulata* (Collin 2000, Richard et al. 2006, Brante et al. 2009). The size of the veliger of *C.aurita*, when liberated in the water column, is ca. 300 μm and comparable to other calyptraeid planktotrophic species (Collin 2003b), but approx. one third smaller than those of *C.fornicata* (Pechenik et al. 1996a, Pechenik et al. 1996b).

4.5 ± 1.3 % of females were infested by the pinnotherid crab *Calyptraeotherespolitus*, which inhabits the mantle cavity of the limpets. This obligatory symbiont to slipper snails (Campos 1999) is also present in *Crepipatella* spp. in the same area. Chaparro et al. (2001b) showed that *Crepipatellaperuviana* (named as *C.fecunda*) females that hosted a pinnotherid crab, which was most probably the same species as the one found in our study, in the incubation space did not deposit eggs during a 12–month study period. However, infestation rate of *C.aurita* was low and most probably did not influence population reproduction.

The transplantation experiment demonstrated that *Calyptraeaaurita* can survive, grow, and reproduce successfully in the shallow subtidal zone, although it was never found at that depth in the field. The vertical distribution of *C.aurita* is discrete and marked by its complete absence in depths of less than 25 m, whereas another calyptraeid species, *Crepipatellaperuviana* (named as *C.fecunda*), dominates the sessile fauna (del Moral and Schories, pers. comm.). Although abundance of *C.peruviana* diminishes with increasing depth, the free space is not used immediately by *C.aurita*. Additional factors than competition for space must explain its absence in shallower water. *C.aurita* does not form continuous belts but shows a patchy distribution. This explains the huge differences in abundance and coverage between the four sample sites, because in the immediate vicinity of the transect lines dense patches of *C.aurita* were always found. Broderip (1834) and Reeve (1859) reported that this species was found at depth between 45–60 fathoms (82–110 m) on shells in sandy mud. As a suspension feeder, filtering phytoplankton and particulate organic matter, *C.aurita* may find sufficient food to develop permanent populations in depths where other grazing gastropods are limited. Its depth distribution is deeper than reported for *Crepidulafornicata* which is known to settle down to 64 m (Orton 1950, Blanchard 1997), *Crepidulaargentina*, 35–50 m (Simone et al. 2000) and *Crepidulaunguiformis*, 70 m (Osorio et al. 2006); however, Cárdenas et al. (2008) reported the presence of *Crepipatelladilatata* and *Crepidulaphilippiana* in 70 to 160 m and 252 m depth, respectively, in the northern Patagonia of Chile.

Dense aggregations of calyptraeid species covering up to 100% of the substrate are common. Blanchard (2009) reported that 85% of an analyzed area from 0 to 15 m depth was inhabited by *Crepidulafornicata* making up the highest benthic biomass. In southern Chile *Crepipatella* spp. shows comparable densities. *Calyptraeaaurita* was found in patches covering up to 50 % of the substrate. This value is still high, because phytoplankton availability is markedly reduced (Gieseke, pers. comm.) and resuspended particulate material might be the main food supply. We found up to 1475 ind. m^-2^ with a mean of 743 ± 307 ind. m^-2^ at Caleta Yerbas Buenas. Population levels reported in the Bay of Brest in France for *Crepidulafornicata* are similar although peak concentrations of 4000 to 5000 individuals can be much higher (Dupont 2004, Guerin 2004, Richard 2005). Abundances of C. *fornicata* reported for the German Wadden Sea are, in general, lower (Thieltges et al. 2003), although maximum density is in the same order as that of *C.aurita*.

## Conclusions

Using traditional shell characteristics, *Calyptraeaaurita* bears no resemblance to suggested synonyms among other species. Most probably the species has not been redescribed before due to its depth distribution, which is below the main diving activities in the region. *Calyptraeaaurita* can be easily identified by the spiral channel present in the umbilicus at the inner surface of the shell and by the sculpture with numerous fine radial ribs. In addition the edges of the shell have a regular circular shape. However, we never found empty shells of this species on the beaches or in the intertidal zone, which might be explained by the pronounced depth gradient along the rocky coast of the eastern part of the Reloncaví Sound, also the material type of the species was dredged at 82–110 m. In contrast to the Atlantic coast of South America no detailed revision of the genus *Calyptraea* (s. s.) has been undertaken.

## Supplementary Material

XML Treatment for Calyptraea aurita
